# A Case of Pulmonary-Renal Syndrome Leading to the Diagnosis of Legionnaires' Disease

**DOI:** 10.1155/2016/4250819

**Published:** 2016-11-23

**Authors:** Erasmia Sabani, Pantelis A. Sarafidis, Antonios Lazaridis, Theodora Kouloukourgiotou, Konstantinos Stylianou, Afroditi Pantzaki, Aikaterini Papagianni, Georgios Efstratiadis

**Affiliations:** ^1^Department of Nephrology, Hippokration Hospital, Aristotle University of Thessaloniki, 49 Konstantinoupoleos Street, 54642 Thessaloniki, Greece; ^2^Second Propaedeutic Department of Internal Medicine, Hippokration Hospital, Aristotle University of Thessaloniki, 49 Konstantinoupoleos Street, 54642 Thessaloniki, Greece; ^3^Department of Nephrology, University Hospital of Heraklion, Andrea Kalokerinou Street, 71500 Heraklion, Greece; ^4^Department of Pathology, Hippokration Hospital, Aristotle University of Thessaloniki, 49 Konstantinoupoleos Street, 54642 Thessaloniki, Greece

## Abstract

We report a case of a 51-year-old Caucasian man referred at our department due to acute renal failure (ARF) complicating respiratory failure during hospitalization in a regional hospital. The patient was previously started on steroids due to the suspicion of rapidly progressive glomerulonephritis (RPGN) in the context of Goodpasture syndrome. However, clinical and laboratory findings did not support this diagnosis; instead a careful evaluation limited differential diagnosis of the renal insult to acute tubular necrosis or acute interstitial nephritis (AIN) following respiratory infection. With lung function fully improved but renal function not recovering, a renal biopsy revealed AIN, a finding leading to further diagnostic testing and finally to the diagnosis of Legionnaires' disease as a cause of this patient's pulmonary-renal syndrome. The management consisted of progressive tapering of oral steroids associated with full recovery of the patient's renal function. This is a rare case of Legionnaires' disease causing immune-mediated AIN and highlights the possibility of Legionella infection as a cause of pulmonary-renal syndrome.

## 1. Introduction

The term pulmonary-renal syndrome includes two categories of clinical entities: first, Goodpasture syndrome which includes a group of autoimmune disorders, namely, Goodpasture disease and systemic vasculitides, and, second, a group of nonautoimmune disorders of various aetiologies that can cause acute lung and renal failure. Legionnaires' disease caused by* Legionella pneumophila* is a common cause of severe pneumonia that can rarely affect the kidney with different mechanisms. We report a case of acute pulmonary and renal failure that was misdiagnosed for Goodpasture's syndrome, whereas a careful clinical and laboratory evaluation ended with the diagnosis of Legionnaires' disease complicated by acute interstitial nephritis (AIN) and full recovery of the patient's renal function with simple therapeutic measures.

## 2. Case Presentation

A 51-year-old man was referred to our tertiary Department of Nephrology from a regional hospital, due to acute renal failure (ARF) following a severe lung infection. His recent medical history started 15 days ago, at the beginning of January, when he presented to the emergency room (ER) of the regional hospital with fever (up to 40°C), chills, and productive cough for three days prior to his admission. He had no history of chronic diseases or drug abuse. His kidney function was normal (serum creatinine 0.95 mg/dL) a year before. He was a smoker and he worked as a cook in a local hotel from April to October each year.

**Figure 1:**
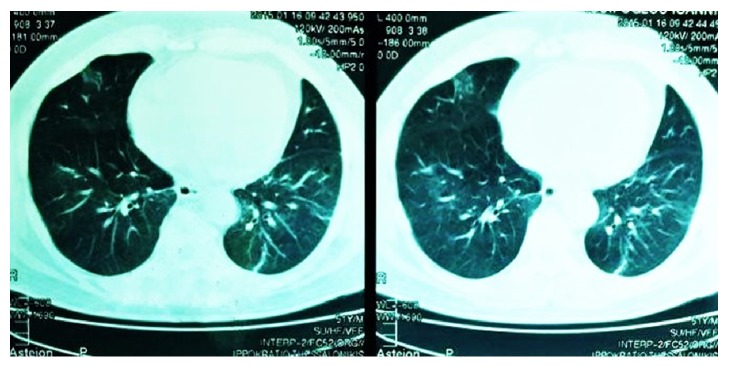
Computed tomography of the lungs showing diffuse bilateral opacities in the lower lobes.

At presentation to the ER he was febrile (39.2°C), blood pressure (BP) was 135/79 mmHg, and pulse was 76 beats per minute. His oxygen saturation was 91.6% in room air, with blood gas showing pH = 7.53, pO_2_ = 55 mmHg, pCO_2_ = 32 mmHg, and FiO_2_ = 21%. Auscultation of the chest revealed diffuse bilateral crackles. The rest of the clinical examination was normal. A chest radiograph showed bilateral lower lung infiltrates and a computed tomography (CT) revealed diffuse bilateral opacities in the lower lobes (Figure 1).

Laboratory tests on admission showed normal white blood cells (4230/mm^3^, 89% neutrophils, 7% lymphocytes, and 1.7% monocytes), hematocrit at 33.3%, hyponatremia (Na 128 mmol/L), markedly elevated C-reactive protein levels (60 mg/dL), and Erythrocyte Sedimentation Rate (90 mm/min). The rest of his work-up showed serum creatinine 1.23 mg/dL, blood urea 57 mg/dL, AST 80 IU, ALT 44 IU, CPK 523 IU/L, and LDH 402 IU/L (Table 1). Urinalysis showed 2+ proteinuria, while sediment showed 1-2 erythrocytes and 1-2 leukocytes per high-power field. The patients' blood cultures, viral serologies (EBV, CMV, HCV, HBV, and HIV), and skin test for tuberculosis (PPD test) were negative.

The patient was started on combined antibiotic treatment with piperacillin-tazobactam, moxifloxacin, and oseltamivir with considerable improvement of his fever, respiratory symptoms, signs, and radiological findings over the next few days. However, his renal function gradually declined with serum creatinine increasing to 2.52 mg/dL and blood urea to 91 mg/dL at Day 3. Due to the combined pulmonary and renal disease the suspicion of rapidly progressive glomerulonephritis (RPGN) due to systemic vasculitis was hypothesized and pulse steroid treatment at a dose of 500 mg of intravenous methylprednisolone daily was initiated at Day 4, and the patient was referred to our department at Day 8, for further evaluation and treatment.

At presentation in our department, the patient was afebrile, BP was at 158/80 mmHg, pulse was 71 beats per minute, respiratory rate was at 25 breaths/minute, and oxygen saturation was 94% in room air. Blood gas showed pH = 7.35, pO_2_ = 80.5 mmHg, pCO_2_ = 20.7 mmHg, HCO_3_ = 11.1 mmol/L, ABE = −13.1 mmol/L, and sO_2_ = 94% while auscultation revealed crackles at the lower lobes of his lungs. He had pitting peripheral edema while his urinary output was normal. The rest of the clinical examination was unremarkable. His laboratory testing showed elevated serum creatinine 3.42 mg/dL and urea 165 mg/dL. However, urine analysis was once again normal (1-2 erythrocytes and 0–2 leukocytes, no casts, and no abnormal erythrocytes) and proteinuria was at 800 mg/24 h. A renal ultrasound showed slightly enlarged kidneys (right, 11.5 cm and left, 12.4 cm) with increased width of cortex (1.5 cm and 2.6 cm). Although the diagnosis of pulmonary and renal failure were evident, due to the absence of nephritic syndrome and the very short period of renal function decline, the diagnosis of RPGN and, therefore, Goodpasture syndrome of any cause could not be supported. Further, the patient had no clinical or radiology signs of lung haemorrhage. At this point the differential diagnosis for his acute kidney injury (AKI) included prerenal AKI due to decrease in renal blood supply at his previous hospitalization (not supported by the referral letter information) and renal AKI due to acute tubular necrosis (ATN) induced by drug agents or AIN caused by drugs or microbial agents. For these reasons the pulse methylprednisolone treatment was stopped when 3 gr in total was reached and followed by oral methylprednisolone at a dose of 48 mg daily with fast tapering. Piperacillin-tazobactam was stopped at Day 9 as it is an agent that could cause ATN or AIN. The patient was supported with conservative measures (including diuretics), with respiratory symptoms, signs, and BP normalizing over the next 2-3 days and his peripheral edema gradually resolving. Over the next days serum immunology testing (IgG, IgA, IgM, C3, C4, ANA, ANCA, anti-dsDNA, anti-GBM, rheumatoid factor, cryoglobulins, serum protein electrophoresis, and immunoglobulin-free *κ*/*λ* chains) was negative, confirming the differential diagnosis.

**Figure 2:**
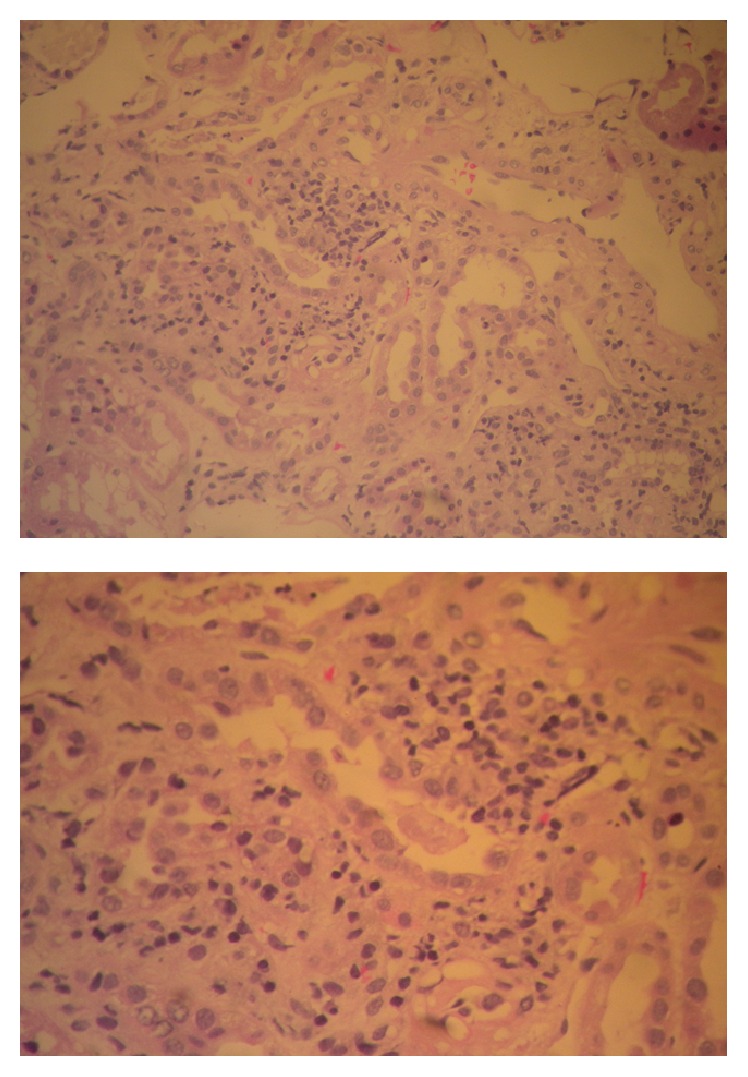
Renal biopsy: findings from light microscopy showing evidence of AIN with interstitial edema and inflammatory peritubular infiltrations composed mainly of lymphocytes. The glomeruli have evidence of nonspecific mild mesangial enlargement, with no abnormalities of the glomerular basement membrane.

With the patient at stable condition, renal function slightly improved but reached a plateau between Day 10 and Day 12 (serum creatinine 3.52, urea 188 mg/dL). On this basis, moxifloxacin was also interrupted at Day 13, as it could also be a causative factor for AIN, and an ultrasound guided biopsy was performed to diagnose AKI of renal origin at Day 15. Light microscopy showed evidence of acute tubulointerstitial nephritis with interstitial edema and inflammatory peritubular infiltrations composed mainly of lymphocytes. The glomeruli had evidence of nonspecific mild mesangial enlargement, with no abnormalities of the glomerular basement membrane (Figure 2). Immunofluorescence showed no evidence of immune deposits. On this basis further testing for Hantaan virus and* Legionella pneumophila* antigen was ordered, while a more detailed record of the patient's history revealed that the patient had recently worked for two nights as a cook in his hotel, which opened only for a weekend over the holiday season. On this basis, the diagnosis of AIN due to* Legionella pneumophila* was confirmed by a positive antigen test at the urine sample returning at Day 21.

After the biopsy the patient was in a stable condition, with his renal function progressively improving with oral steroid use tapered to a dose of 4 mg per week. He was discharged from our hospital at Day 22 with his serum creatinine at 1.87 mg/dL at 28 mg methylprednisolone daily (Table 2). He was closely followed up in the Outpatient Department with serum creatinine at 1.53 mg/dL at 1 month after discharge and returned to normal (0.88 mg/dL) at two months after discharge (Table 2) with cessation of steroid treatment.

## 3. Discussion

The term pulmonary-renal or lung-kidney syndrome refers to two major different clinical entities. The first major clinical category includes Goodpasture syndrome which is a term used to describe a group of autoimmune disorders characterized by the combination of diffuse alveolar haemorrhage (DAH) and rapidly progressive glomerulonephritis. This includes Goodpasture disease (also known as anti-GBM disease) and a group of systemic vasculitides such as Wegener's disease, systemic lupus erythematosus, Churg-Strauss syndrome, microscopic polyangiitis, essential mixed cryoglobulinemia, and rheumatoid vasculitis where ANCA (either c-ANCA or p-ANCA) are usually detected [1].

The second major clinical category includes severe heart failure with acute pulmonary oedema combined with ARF, Acute Respiratory Distress Syndrome (ARDS) with renal failure in the case of multiorgan failure, paraquat poisoning, renal vein-inferior vena cava thrombosis with pulmonary emboli and numerous pathogens that can cause pneumonia and ARF such as* Streptococcus pneumoniae*,* Leptospira*,* Legionella pneumophila*,* Salmonella*,* Yersinia*, Corynebacteria,* Brucella melitensis*, and* Hantavirus*, and other mainly opportunistic microorganisms such as EBV, CMV,* Candida*, and* Mycobacterium tuberculosis*.

The differential diagnosis in the case of pulmonary-renal syndrome is highly important. Early recognition of Goodpasture syndrome is crucial as the prognosis for the recovery of kidney function depends mainly on the extent of the initial injury and thus early aggressive therapy including corticosteroids, cyclophosphamide, and plasmapheresis is warranted [2]. The diagnostic approach includes careful clinical evaluation of the patient's medical history, physical examination, radiological imaging with CT in order to detect lung involvement, biochemical evaluation including serum urea and creatinine levels, and urine analysis to detect proteinuria, haematuria, or active urine sediment including white cell or red cell casts and targeted laboratory exams including immunological assays (especially in the presence of pulmonary haemorrhage where the suspicion of systemic vasculitis is placed high).

Our patient presented with ARF from the third day of his hospitalization, without evidence of extrarenal involvement from the radiological imaging. Immunological testing (ANA, c-ANCA, p-ANCA, and anti-GBM) was negative and the urine analysis revealed a blunt urine specimen. Therefore, the suspicion of Goodpasture syndrome could not be supported. The differential diagnosis of AKI at that point included AIN due to drug use or infection and ATN induced by volume depletion and hypotension. There was no clinical evidence of hemodynamic instability to support the suspicion of prerenal AKI and piperacillin-tazobactam was stopped without further improvement of renal function. Thus, a biopsy was performed in order to place the final diagnosis and decide further treatment with findings being suggestive of AIN. With the urine testing for* Legionella* returning positive and no other predisposing factors, the diagnosis of AIN due to* Legionella* infection was made.

Legionnaires' disease was first described in 1976 at an outbreak that erupted among participants of the 58th Congress of the American Legion in Philadelphia. Since then it has been well recognized as one of the most common causes of severe community acquired pneumonia in Europe [3]. It is caused by the Gram negative bacteria* Legionella* with* L. pneumophila* type I being the most common serogroup involved. The disease can clinically present as two separate entities:* Legionella pneumonia* with other system involvement and Pontiac fever (a nonpneumonic flu-like disease). It is transmitted to humans by inhalation of infected aerosol; recently a case of probable person-to-person transmission has been also described [4]. Patient risk factors associated with* Legionella* infection include age >50 years, smoking, chronic lung disease, glucocorticoid administration and transplantation, or illnesses associated with immunosuppression [5].* Legionella* can affect multiple organ systems including the gastrointestinal tract and the central nervous system but only a few cases of renal involvement have been described, the spectrum of which ranges from mild elevation of serum creatinine levels to acute anuric renal failure requiring dialysis [6].

Unfortunately, there are no specific clinical or laboratory tests confirming the diagnosis. The urine antigen test for* Legionella* is highly specific and can be positive for days even after antibiotic treatment; yet it detects only serotype I and consequently a negative result will not exclude the diagnosis. Nevertheless, the suspicion of Legionnaires' disease should be high when there is a case of acute severe pneumonia in an epidemic context, with bilateral pulmonary involvement, hyponatremia, elevated CPK levels, and extrapulmonary symptoms especially including the gastrointestinal tract.

The pathogenesis of renal involvement remains unknown and several mechanisms have been proposed including toxemia, drug toxicity, hypotension, direct nephrotoxicity of the microorganism, and immune-mediated nephrotoxicity [7, 8]. Most renal biopsies performed in patients with confirmed diagnosis of Legionnaires' disease and renal involvement include findings of tubulointerstitial nephritis and/or ATN with a favorable response to corticosteroid treatment and antibiotics [8]. In 1978, Relman et al. reported the first case of AIN in a patient with pneumonia due to Legionnaires' disease [9], and two other cases followed [7, 10]. In 1987, a case of Legionnaires' disease affecting the kidneys but not the lungs was described [11] and, later, Verhaeverbeke et al. described a case of* Legionella*-induced AIN that improved after temporary hemodialysis and antibiotic but not corticosteroid administration [12]. More recently, Nishitarumizu et al. reviewed 45 cases of ARF in patients with Legionnaires' disease, among which 15 had a renal biopsy performed. The results of the renal biopsies demonstrated 5 cases of AIN, 6 cases of ATN, 1 case of crescentic glomerulonephritis, 1 case of proliferative mesangial glomerulonephritis, and 2 cases of pyelonephritis [13].

Our case highlights the fact that pulmonary-renal syndrome includes both autoimmune and nonautoimmune disorders and, thus, a patient presenting with respiratory failure and AKI should be investigated in both directions to establish the diagnosis. In this case, the initial assumption of Goodpasture's syndrome was not confirmed by either the lung or the kidney findings; in contrast, a proper diagnostic approach revealed the presence of pneumonia and AIN. This, with reevaluation of the patient occupational history led to the diagnosis of Legionnaires' disease, which was not considered in initial presentation. Based on the biopsy findings and the different course of pulmonary and renal injuries from* Legionella*, corticosteroid treatment led to a favorable response, as would happen in most patients with immune-mediated AIN. Overall, Legionnaires' disease is an uncommon cause of pulmonary-renal syndrome but should be included in the differential diagnosis and properly treated, to spare the patients from irreversible kidney damage.

## Figures and Tables

**Table 1:** Changes in laboratory values during the clinical course of two hospitalizations.

	1st admission Day 0	Day 3	Day 7	2nd admission Day 8	Day 13	Day 22	Reference values
WBC (K/*μ*L)	4230	5640	11910	14790	14600	4600	3.8–10.5
Ht/Hb (%, gr/dL)	33.3/11.6	27.8/9.8	29.5/10	29.8/10.36	30.8/10.4	36/12	40–52/14–18
PLTs (K/*μ*L)	147000	155000	303000	338000	245000	80000	150–450.000
SGOT/AST (U/L)	80	82		18	8	6	10–37
SGPT/ALT (U/L)	44	33		30	25	28	10–45
LDH (U/L)	455	481		359	375	240	<248
CPK (U/L)	656	251		34	50	25	<170
ALP (U/L)	30			31	45	49	30–120
*γ*GT/GTT (U/L)	25			33	34	32	<55
Total proteins/albumin (gr/dL)	5.5/2.7			5.7/2.8	6.3/3.1	6.7/3.5	6.6–8.3/3.5–5.2
Urea (mg/dL)	57	91	175	165	184	133	10–43
Creatinine (mg/dL)	1.23	2.52	4.72	3.42	3.84	1.87	0.81–1.2
Potassium (mEq/L)	3.8	3.4	3.5	3.9	4.1	4.2	
Sodium (mEq/L)	128	137	147	140	143	141	136–145
Calcium (mg/dL)		7.9		7.8	7.2	8.3	
Phosphorus (mg/dL)				5	5.2	3.6	
ESR (mm/h)	44			44			<20
CRP (mg/dL)		5.02		4.57	1.47	0.50	<0.5

**Table 2:** Full blood count, kidney function, and steroid dose on admission and after discharge.

	Day of admission	Day of discharge	1 month later	2 months later
WBC (K/*μ*L)	14.790	16.200	17.070	9.220
Hct (%)	29.8	36	37.7	39.2
Urea (mg/dL)	165	133	83	33
Creatinine (mg/dL)	3.42	1.87	1.53	0.88
Methylprednisolone	36 mg/24 h	28 mg/24 h	4 mg/24 h	
